# Motivational system modulates brain responses during exploratory decision-making

**DOI:** 10.1038/s41598-021-95311-0

**Published:** 2021-08-04

**Authors:** Chia-Wei Li, Carol Yeh-Yun Lin, Ting-Ting Chang, Nai-Shing Yen, Danchi Tan

**Affiliations:** 1 Department of Radiology, Wan Fang Hospital, Taipei Medical University, Taipei, Taiwan, ROC; 2 Department of Business Administration, National Chengchi University, Taipei, Taiwan, ROC; 3 Research Center for Mind, Brain & Learning, National Chengchi University, Taipei, Taiwan, ROC; 4 Department of Psychology, National Chengchi University, Taipei, Taiwan, ROC; 5 Department of International Business, National Chengchi University, Taipei, Taiwan, ROC

**Keywords:** Neuroscience, Psychology

## Abstract

Managers face risk in explorative decision-making and those who are better at such decisions can achieve future viability. To understand what makes a manager effective at explorative decision-making requires an analysis of the manager’s motivational characteristics. The behavioral activation/inhibition system (BAS/BIS), fitting the motivational orientation of “approach” or “avoidance,” can affect individual decision-making. However, very little is known about the neural correlates of BAS/BIS orientation and their interrelationship with the mental activity during explorative decision-making. We conducted an fMRI study on 111 potential managers to investigate how the brain responses of explorative decision-making interact with BAS/BIS. Participants were separated into high- and low-performance groups based on the median exploration-score. The low-performance group showed significantly higher BAS than that of the high-performance group, and its BAS had significant negative association with neural networks related to reward-seeking during explorative decision-making. Moreover, the BIS of the low-performance group was negatively correlated with the activation of cerebral regions responding to risk-choice during explorative decision-making. Our finding showed that BAS/BIS was associated with the brain activation during explorative decision-making only in the low-performance group. This study contributed to the understanding of the micro-foundations of strategically relevant decision-making and has an implication for management development.

## Introduction

Organizations need both exploitation and exploration in order to survive and prosper in changing environments^1–3^. Exploitation involves maintaining or improving existing processes^4^. Exploration involves a pursuit of new knowledge that often leads to radical innovation. It thus prevents organizations from becoming trapped in suboptimal stable equilibria^5^. However, compared to exploitation, exploration yields return that are often lower^6^, systematically less certain, and more remote in time. Given that exploration is needed for organizations to achieve “future viability”^7^, it is important to understand why some individuals are good at making explorative decisions while others are not.

Researchers suggest that an individual’s cognitive and motivational characteristics may influence how they make explorative decisions^4,8^. For example, it has been found that a person with an entrepreneurial mind-set values uncertainty in the marketplace and continuously explores opportunities with the potential to lead to important innovations^9^. That is, entrepreneurs embrace uncertainty and their exploration begins with the motivation to discover new opportunities^10^.

In this paper, we investigate why some individuals showed low or high performance at making explorative decision and how the performances are related to their motivational systems. We draw on the motivational scale of the behavioral activation system (BAS) and the behavioral inhibition system (BIS) developed by Carvers and White in 1994. BAS/BIS has been widely used in education, psychology and organizational behavior research, though less so in entrepreneurship studies^11^. It is a commonly used instrument in assessing an individual’s approach or avoidance behavior, as BAS examines appetitive motives to move toward something desired, and BIS examines aversive motives to move away from something unpleasant^11^. Prior research shows that BAS is correlated with both enterprising interest and confidence, whereas BIS is negatively correlated with indifferent responses, realistic interests, and approach under uncertainty^12^. Accordingly, an individual’s motivation orientation of approach or avoidance may influence their exploration and exploitation decision-making and consequential performance. Specifically, people characterized by approach (BAS) motivation may be keen on exploring new information (exploration), while those characterized by avoidance (BIS) motivation may be satisfied with current rewards and avoid trying new ways of doing things (exploitation). Therefore, the motivational orientation of “approach” (BAS) or “avoidance” (BIS) may be used to predict what type of person is a better fit for a particular type of task or decision context. That is, investigating the relationship between BAS or BIS orientation and exploration performance may serve as a good point of reference for strategic talent deployment in organizations.

Functional MRI (fMRI) enables researchers to investigate the profile of brain responses by depicting the hemodynamic response associated with brain activity invasively. This technique has been used to investigate mental motivational decision-making and management^4,6,13,14^. Daw et al.^13^ suggest that exploitation requires the engagement of the neural circuits associated with the self-reward system whereas exploration involves a higher-level of the cognitive control brain network^13^. Krug et al. used fMRI to investigate mental activity during decision-making under uncertainty^14^. They report a network comprised of the supplementary motor cortex, anterior cingulate cortex, right prefrontal cortex, insula, and precuneus. The above circuits are correlated to the uncertainty acceptance condition while right middle frontal cortex is correlated to the uncertainty avoidance condition. Laureiro-Martinez et al.^4^. are the pioneers in applying fMRI technique in management research and in investigating the relationship of attention control and decision-making performance from the perspective of the exploitation-exploration dilemma^6^. In their 2014 paper, they used fMRI and the “four-armed bandit” task to investigate the efficiency of strategy between managers and entrepreneurs^4^. Compared with managers, entrepreneurs exhibited higher decision-making efficiency with stronger activity in regions of the frontopolar cortex associated with explorative choice. Activity across seven brain networks previously linked to exploit/explore tradeoffs explained individual differences in choice efficiency. In their follow-up study^6^, they used fMRI to examine 63 expert decision makers with respect to their cognitive processes and found that exploitation activates regions associated with reward seeking which track and evaluate the value of current choices, while exploration relies on brain regions associated with attentional control which track the value of alternative choices. These previous efforts have identified the activity of fronto-insular- parietal networks during exploration. However, whether and how these networks are associated with approach and avoidance personality characteristics are still unknown.

In this study, we aimed to investigate why some individuals showed high performance at making explorative decisions and to find out the relationship between BAS/BIS and the individual’s mental activity stimulated during explorative decision-making in high- and low-performance groups. For this purpose, we used fMRI to assess 111 students (potential managers) from a renowned university in a “four-armed bandit” task and to examine the correlation between individual’s mental activity and BAS/BIS during exploitative and explorative decision-making. To achieve higher scores in this task, participants were instructed to choose between foreseeable stable rewards (exploitation) or to bet on a long-term larger gain under uncertainty (exploration). We first investigate the mental activation during exploitation and exploration decisions-making, predicting that exploitation will be associated with the self-reward system whereas exploration will involve the cognitive control brain network, according to Daw et al.^13^ study. We then investigate how cerebral circuits interact with BAS/BIS, expecting that low BAS would associate with exploitation, whereas low BIS would show association with explorative decision making, based on previous study that BAS personality tends to explore new information and BIS tend to be satisfied with current rewards and taking exploitation approach. We further investigate how the mental activation correlated with the BAS/BIS scores during explorative decision making.

## Results

### Overall brain responses

**Figure 1:**
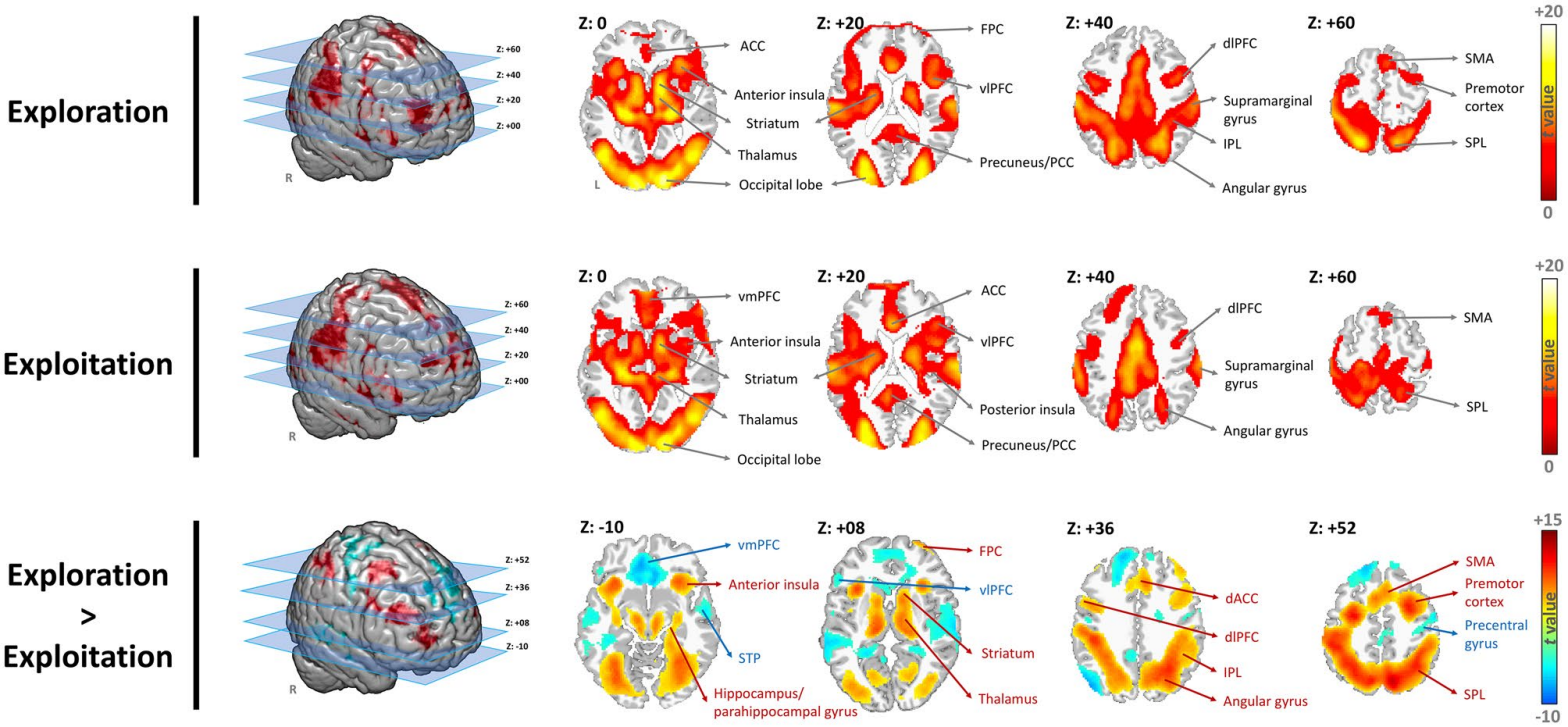
Activated brain areas while exploration and exploitation were performed. The threshold was set at Alpha-shim corrected p < 0.05 for multiple comparisons (*ACC* anterior cingulate cortex, *dACC* dorsal anterior cingulate cortex, *dlPFC* dorsolateral prefrontal cortex, *FPC* frontopolar cortex, *IPL* inferior parietal lobule, *PCC* posterior cingulate cortex, *SMA* supplementary motor area, *SPL* superior parietal lobule, *STP* superior temporal pole, *vlPFC* ventrolateral prefrontal cortex, *vmPFC* ventromedial prefrontal cortex).

Our first step is to investigate overall brain responses of exploitation and exploration decision-making. During each trial, the individual analysis was applied based on the events of explorative decision making (choose a new option in the hope of a higher payoff) and exploitative decision making (choose an uncertain but familiar option), according to the interpretation of Daw et al.^13^. At the group level, both exploitation and explorative decision-making were associated with the activation of the middle frontal gyrus (MFG), dorsolateral prefrontal cortex (dlPFC), ventrolateral prefrontal cortex (vlPFC), supplementary motor area (SMA), ventromedial prefrontal cortex (vmPFC), inferior parietal lobule (IPL), superior parietal lobule (SPL), vision-related areas, hippocampus, striatum, middle temporal gyrus (MTG), supramarginal gyrus, anterior insula, anterior cingulate cortex (ACC), and sensorimotor areas (Fig. 1 and Table 1). Compared with exploitation, explorative decision-making was associated with higher brain activity of the PCC, premotor cortex, SMA, IPL/SPL, dlPFC, striatum, thalamus, frontopolar cortex (FPC), and angular gyrus. In contrast, exploitation was associated with greater activations within vmPFC, vlPFC, and precentral gyrus relative to exploration. The activation maps during exploitative/explorative decision-making were used as masks for further data analysis.

**Table 1:** The activation during explorative and exploitative decision-making.

Volume information	Peak coordinates			*t* value	Cluster (Voxels)
	x	y	z		
**Exploration**					
Fusiform gyrus, middle temporal gyrus	30	− 72	− 10	20.36	89,547
Lingual gyrus	− 12	− 90	− 10	18.62	
Calcarine sulcus	14	− 92	− 4	18.34	
Hippocampus	− 24	− 26	− 8	18.06	
Superior occipital gyrus	18	− 98	6	17.73	
Middle occipital gyrus, Angular gyrus	− 26	− 92	10	17.51	
Thalamus, striatum	20	− 24	2	17.33	
Precuneus, cuneus	22	− 92	12	16.91	
Inferior parietal lobule, precuneus	32	− 52	50	15.26	
Supramarginal gyrus	− 60	− 22	16	15.06	
Anterior insula, ventromedial prefrontal cortex	36	22	− 4	14.66	
Precentral gyrus, premotor cortex, supplementary motor area, dorsolateral prefrontal cortex	48	6	28	14.66	
Dorsal anterior cingulate cortex	− 2	24	26	12.33	
**Exploitation**					
Fusiform gyrus	− 22	− 84	− 10	19.45	90,107
Hippocampus	− 24	− 26	− 8	17.45	
Superior occipital gyrus	24	− 94	14	17.23	
Middle occipital gyrus, Angular gyrus	− 26	− 90	10	17.19	
Calcarine sulcus	14	− 92	− 4	16.95	
Middle temporal gyrus	− 48	− 68	2	14.97	
Supramarginal gyrus, dorsolateral prefrontal cortex	64	− 18	22	14.97	
Ventrolateral prefrontal cortex	48	34	− 4	13.80	
Posterior cingulate/precuneus	− 6	− 46	36	13.66	
Dorsal anterior cingulate cortex, ventromedial prefrontal cortex	0	30	16	13.46	
Posterior insula	− 32	− 6	10	12.47	
Pons	2	− 38	− 34	4.80	99
**Exploration > exploitation**					
Precuneus/posterior cingulate cortex	12	− 68	50	12.43	46,342
	− 8	− 68	50	10.20	
Precentral gyrus, postcentral gyrus	− 38	− 34	44	11.99	
	− 48	− 34	54	10.14	
Inferior parietal lobule	32	− 50	46	10.89	
	− 32	− 52	48	10.81	
Premotor cortex, dorsolateral prefrontal cortex	− 26	− 6	52	10.48	
	28	2	50	10.30	
Superior parietal lobule	− 20	− 66	58	10.36	
Angular gyrus	32	− 58	42	10.23	
Anterior insula, striatum, hippocampus	− 32	22	− 2	9.48	
	32	24	0	9.16	
Frontopolar cortex	− 30	60	20	5.06	232
Posterior cingulate cortex	6	− 26	28	4.96	301
Inferior temporal gyrus	− 32	6	− 32	4.36	91
**Exploitation > exploration**					
Angular gyrus	− 48	− 76	34	8.42	5643
Middle occipital gyrus	− 40	− 80	42	7.20	
Middle temporal gyrus	− 64	− 36	4	6.72	
Hippocampus (L), midbrain	− 22	− 46	10	6.37	
	− 24	− 16	− 22	5.41	
Inferior temporal gyrus	− 60	− 8	− 22	5.64	
Middle cingulate cortex	− 12	− 14	28	5.42	
Ventromedial prefrontal cortex	− 4	38	− 12	8.41	6316
	2	34	− 14	7.56	
Anterior cingulate cortex	− 6	24	− 2	7.60	
	8	24	− 10	7.50	
Dorsolateral prefrontal cortex	− 12	46	46	7.36	
Dorsomedial prefrontal cortex	− 2	56	20	6.23	
	2	58	20	6.22	
Postcentral gyrus, precentral gyrus	14	− 36	72	6.78	5863
Superior temporal gyrus/pole	68	− 20	6	6.46	
Paracentral lobule	2	− 28	60	6.32	
	− 4	− 26	66	5.40	
Supplementary motor cortex	6	− 22	60	6.11	
Dorsolateral prefrontal cortex	54	− 6	52	5.82	
Precuneus	− 8	− 40	66	5.78	
Inferior parietal lobule	− 10	− 36	68	5.57	
Premotor cortex	18	− 18	72	5.23	
Caudate tail	20	− 36	20	6.49	445
Ventrolateral prefrontal cortex	− 52	30	4	4.97	290
Precuneus/posterior cingulate cortex	− 4	− 46	38	4.90	361

Statistical significance was thresholded at uncorrected p < 0.001 with a minimum cluster size of 77 voxels, which yielded an overall false positive p < 0.05 as determined using Alpha-Sim for multiple comparisons correction.

### High and low performance groups

**Figure 2:**
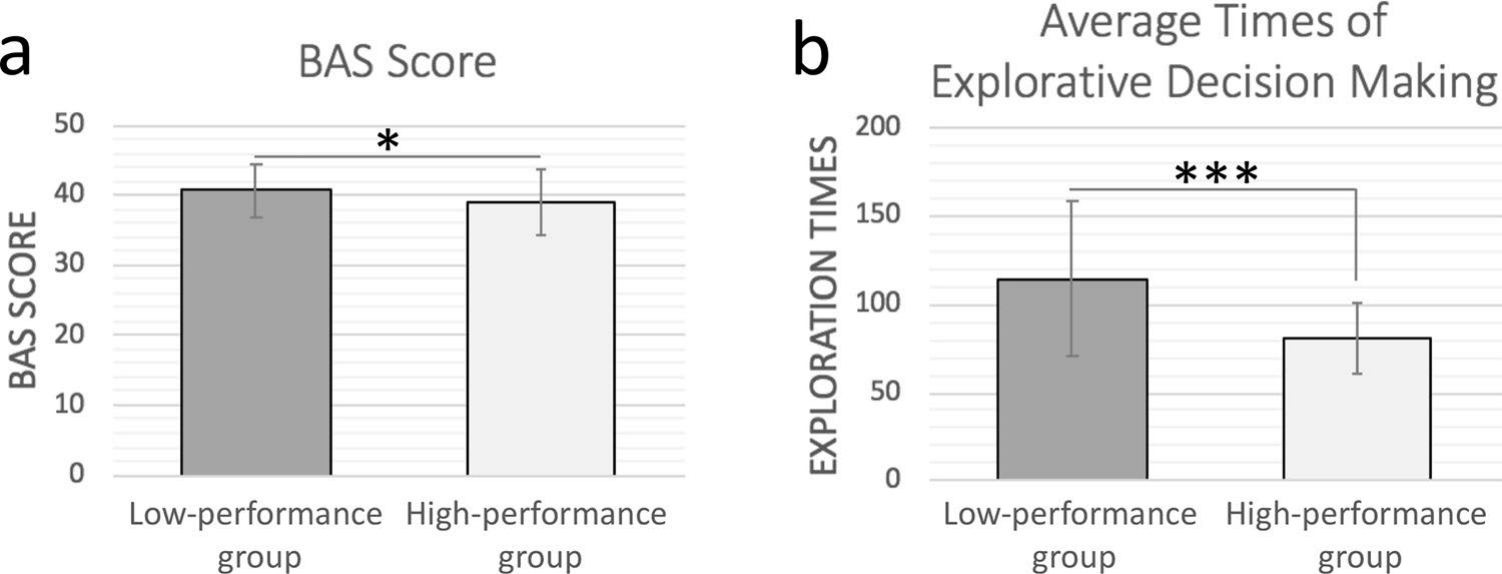
(**a**, **b**) Bar graph of difference between high-/low-performance groups. (**a**) The BAS score of low-performance group was significantly higher than that of high-performance group (*p* < 0.05). (**b**) The exploration times of low-performance group was significantly more than high-performance group’s (*p* < 0.001).

As mentioned previously, we are interested in understanding why people make good or poor explorative decisions. To address this question, we classified all participants into two groups based on their median average score of performance of explorative decision-making, which is 48.86. The *high-performance* group consists of 54 participants whose average explorative scores are higher than 48.86, while the *low-performance* group consists of 57 participants whose explorative scores are lower or equal to 48.86. Noted that the BAS score in the low-performance group was significantly higher than that of the high-performance group (40.7 ± 3.8 of low-performance group and 39.0 ± 4.6 of high-performance group; p < 0.05) (Fig. 2a). The numbers (frequency) of explorative decision-making in the low-performance group were also significantly higher than those in the high-performance group (114.6 ± 44.4 of low-performance group and 80.7 ± 19.6 of high-performance group; p < 0.001) (Fig. 2b). In addition, the BAS scores of the low-performance group were positively correlated to the numbers of explorative decision-making (r = 0.27; p < 0.05), while the BAS scores of the high-performance group did not have this pattern. The BIS scores of the two groups showed no significant differences with the numbers of explorative decision-making.

### Mental activities correlate with behavior results

**Figure 3:**
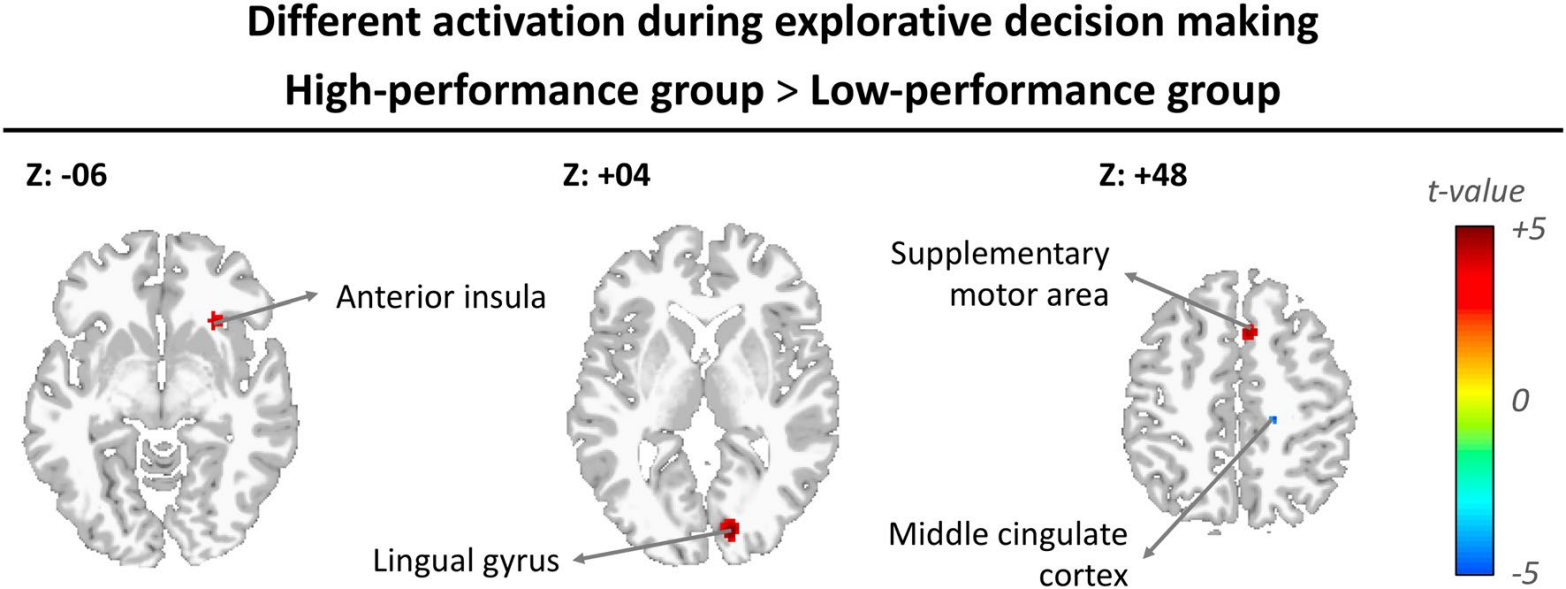
Significant difference between high- and low-performance groups during explorative decision-making. The threshold was set at Alpha-shim corrected p < 0.05 for multiple comparison.

In parallel with behavioral results, brain response profiles of the high and low performance groups during explorative decision making were then examined. Compared with the low-performance group, the high-performance group showed enhanced activity in the anterior insula, SMA, and lingual gyrus (Fig. 3 and Table 2), as well as reduced activity in the middle cingulate cortex during explorative decision-making.

**Table 2:** The significant difference between high- and low-performance groups during explorative decision-making.

Volume information	Peak coordinates			*t* value	Cluster (Voxels)
	x	y	z		
**High-performance group > low-performance group**					
Lingual gyrus	14	− 88	2	3.91	80
Supplementary motor area	6	18	48	3.62	53
Anterior insula	28	20	− 10	3.35	53
**Low-performance group > high-performance group**					
Middle cingulate cortex	20	− 26	48	3.80	49

Statistical significance was thresholded at uncorrected p < 0.003 with a minimum cluster size of 50 voxels, which yielded an overall false positive p < 0.05 as determined using Alpha-Sim for multiple comparisons correction.

**Figure 4:**
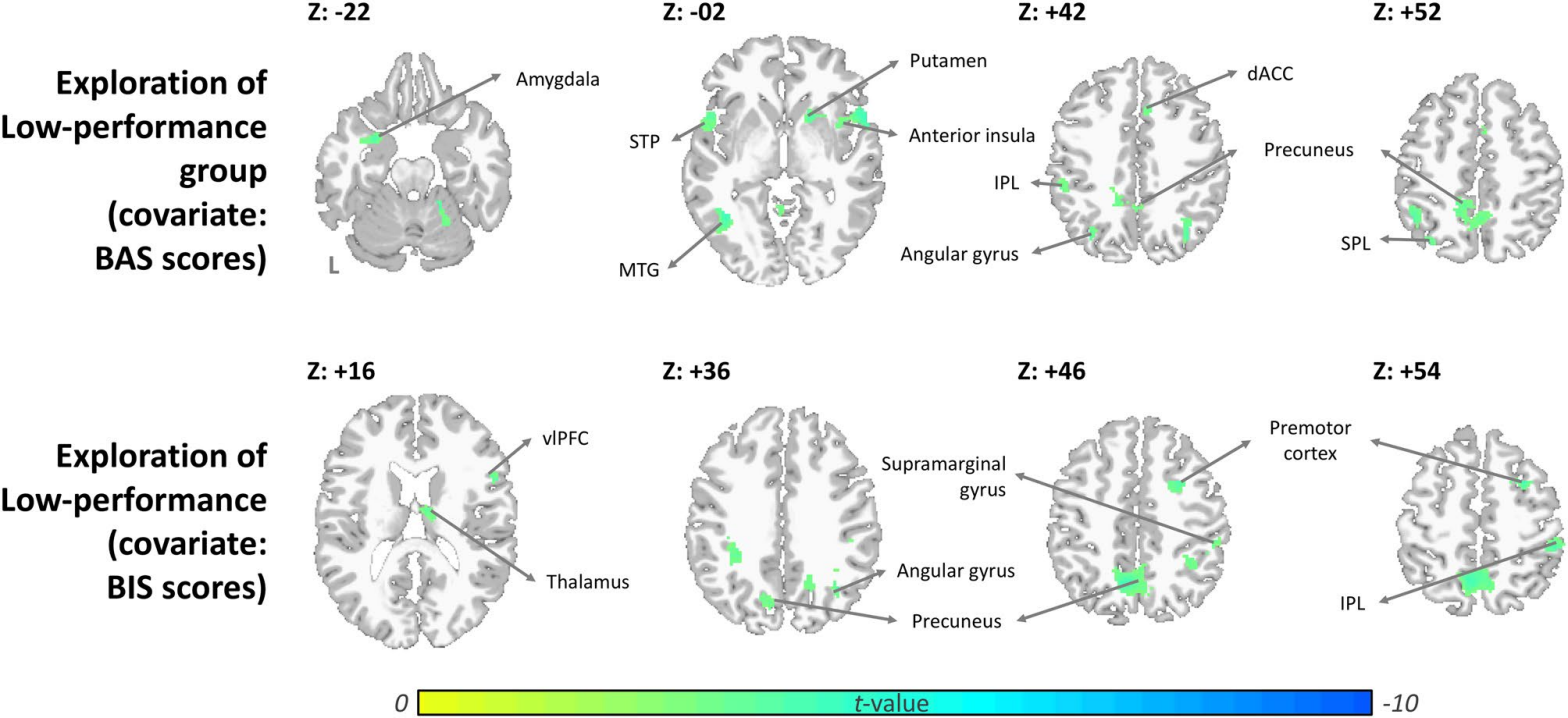
Significant correlation between BAS/BIS score and neural activation during explorative decision-making. The threshold was set at Alpha-shim corrected *p* < 0.05 for multiple comparison. (*ACC* anterior cingulate cortex, *dACC* dorsal anterior cingulate cortex, *dlPFC* dorsolateral prefrontal cortex, *FPC* frontopolar cortex, *IPL* inferior parietal lobule, *PCC* posterior cingulate cortex, *SMA* supplementary motor area, *SPL* superior parietal lobule, *STP* superior temporal pole, *vlPFC* ventrolateral prefrontal cortex, *vmPFC* ventromedial prefrontal cortex).

Afterwards, we investigated how BAS and BIS correlate with brain responses in the high- and low-performance groups. In the low-performances group, the BAS score has a negative linear association with the activity of the anterior insula, MTG, putamen, dACC, IPL/SPL, superior temporal pole (STP), angular gyrus, precuneus, and amygdala (Fig. 4 and Table 3) during explorative decision-making; besides, the BIS score showed a negative linear correlation with the activity of the premotor cortex, supramarginal gyrus, thalamus, IPL, vlPFC, angular gyrus, and precuneus. By contrast, neither BAS nor BIS scores showed a linear association with cerebral activity in the high-performance group during explorative decision-making.

**Table 3:** The BAS/BIS associated activation during explorative decision-making among 2 groups.

Volume information	Peak coordinates			*t* value	Cluster (Voxels)
	x	y	z		
**Exploration of high-performance group (covariate: BAS scores)**					
None					
**Exploration of high-performance group (covariate: BIS scores)**					
None					
**Exploration of low-performance group (covariate: BAS scores)**					
Middle temporal gyrus	− 40	− 56	2	− 4.71	168
Putamen, caudate	18	14	0	− 4.56	179
Superior temporal pole, superior temporal gyrus	60	10	0	− 4.39	218
Precentral gyrus, premotor cortex	− 18	− 10	74	− 4.34	104
Inferior parietal lobule	− 44	− 52	52	− 4.05	93
	36	− 66	42	− 3.89	72
	− 52	14	− 2	− 3.87	94
	− 54	− 34	44	− 3.46	70
Amygdala, parahippocampal gyrus	− 28	2	− 22	− 3.99	55
	− 18	− 30	− 12	− 3.86	50
Cerebellar lobule IV–VI	20	− 46	− 18	− 3.97	79
	− 4	− 52	2	− 3.40	80
Superior temporal gyrus	− 54	− 10	10	− 3.91	99
Superior temporal pole	− 32	− 70	42	− 3.90	94
Anterior/middle cingulate cortex, supplementary motor area	− 12	− 40	48	− 3.81	370
	− 12	18	32	− 3.57	85
	8	18	44	− 3.47	117
Postcentral gyrus	60	− 16	20	− 3.60	54
**Exploration of low-performance group (covariate: BIS scores)**					
Premotor cortex	30	8	50	− 4.60	157
Precuneus	− 6	− 60	50	− 4.55	768
	22	− 54	32	− 3.70	91
	− 16	− 60	28	− 3.44	73
Middle cingulate	0	− 14	26	− 4.25	187
Thalamus	8	− 10	18	− 3.94	56
Inferior parietal lobule, supramarginal gyrus	50	− 36	52	− 3.94	163
Inferior parietal lobule, angular gyrus	38	− 62	36	− 3.93	181
	− 32	− 42	38	− 3.74	95
Ventrolateral prefrontal cortex	56	10	26	− 3.92	105

Statistical significance was thresholded at uncorrected p < 0.003 with a minimum cluster size of 50 voxels, which yielded an overall false positive p < 0.05 as determined using Alpha-Sim for multiple comparisons correction.

To further check whether there is gender difference on behavioral and imaging data, we did a correlation analysis and neural image analysis. Our findings are presented in Table 4 and the Supplementary Figs. S1, S2, and S3. Table 4 showed that sex has a significant correlation with fMRI performance and exploration score. Figures S1, S2, and S3 showed that the male group had similar activated regions as that of female group, though the more cerebral regions are activated for the female group.

**Table 4:** Means, standard deviation and Pearson correlation matrix for Variables (N = 111).

Variables	Mean	SD	1	2	3	4	5	6
1. Sex			**–**					
2. BIS score	20.51	3.43	0.179	**–**				
3. BAS score	39.88	4.28	0.133	− 0.163	**–**			
4. fMRI performance average score	55.77	1.69	− 0.234*	− 0.152	− 0.203*	**–**		
5. Exploration average score	48.97	1.37	− 0.246**	− 0.296**	− 0.051	0.634**	**–**	
6. Exploitation average score	59.32	0.46	0.102	0.233*	− 0.030	− 0.244**	− 0.389**	**–**

Variable sex is categorical variable.

*p < 0.05.

**p < 0.01.

## Discussion

The motivational orientation of “approach” or “avoidance” can affect individual decision-making. However, very little is known about the neural correlates of BAS/BIS orientation and their interrelationship with the mental activity during explorative decision-making. This study has examined the mental activation during exploitation and exploration decisions-making; and investigated how the mental activation correlate with the BAS/BIS scores of high- and low-performance groups during explorative decision making. We found that the parietal and prefrontal cortical regions showed high activity during explorative/exploitative decision making, and that the BAS/BIS scores had a correlation with the activation of the reward-associated network during explorative decision-making.

In particular, our findings showed that the participants of the low-performance group made explorative decisions more frequently in our “four-armed bandit” task; as an association, their BAS scores were significantly higher than that of the high-performance group. Further, their BAS scores showed a negative linear association with the activation of the reward-associated network, including the MTG, putamen, dACC, STP, and amygdala, during explorative decision-making. Greater activities in the ACC, amygdala, and anterior cerebellar lobules have been reported as associated with increased motivation and involvement of memory in processing the cued reward during reward-anticipation and reward-gain^15^. Scholars proposed that the anterior insula detects salient stimuli and coordinates neural resources^16,17^. The STP is a node of the paralimbic system with strong connectivity with the orbitofrontal cortex, striatum, insula, amygdala, and other emotion-related regions^18,19^. As an associated cortex, the STP enables multisensory integration and plays key roles in cognitive and socioemotional processing. Thus, our finding of negative association with BAS and the reward-seeking network for the low-performance group, suggests that too frequent explorative attempts may become blind trials that lead to low performance. In comparison, the high-performance group, which made fewer explorative attempts, employed more calculated and more effective exploration, and resulted in better performance.

In addition, for the low-performance group during explorative decision-making, the BIS scores showed a negative relationship with the activation of the attention and risk-seeking networks, including the vlPFC, thalamus, precuneus/PCC, and IPL. Tobler et al. indicated the vlPFC showed selective decreased activity with variance in risk averters without variance coding in seven risk seekers^20^. Another study also revealed that the connectivity between the vlPFC, posterior cingulate, and precentral gyrus was enhanced in reward sensitive individuals^21^. Hsu et al. indicated that the vlPFC, precuneus, premotor cortex, precuneus, and angular gyrus were sensitive to the level of ambiguity and risk in behavioral choices^22^. In the low-performance group, our findings implicated that the BIS scores were linearly correlated with the decreased activity of networks responding to risk-choices during explorative decision-making. It is likely that the low performance group sees risky explorative decisions as avoidable cues and reacts to the decision with a decreased risk-choices responding network.

In contrast, the high-performance group showed enhanced activation in the anterior insula, SMA, and lingual gyrus during explorative decision-making compared to the low-performance group. The anterior insula has been reported as a key structure in some models of self-regulation and reward seeking^23–25^, because it detects salient stimuli and coordinates neural resources^16,17^. Previous visual-task studies revealed that the activation of the lingual gyrus was associated with visual attention^26–29^. Spooner et al. indicated a robust relationship in the supplementary motor area between validity difference scores in frontal theta activity and movement-locked gamma oscillations, suggesting modulation of these sensorimotor network gamma responses by attentional reorienting^30^. Zorowitz et al. also reported that pre-SMA are specifically correlated with approach-avoidance conflict during employment of a variable epoch method^31^. Compared with the activation of the low-performance group, our results revealed that the activities of the high-performance group in the anterior insula, SMA, and lingual gyrus increase when driven to pursue rewards and avoid harm during explorative decision-making. Thus, people who employ calculated and controlled in reward seeking behavior achieve better performance when making explorative decision.

Although the focus of our study is to understand how motivational systems moderate brain responses during explorative decision making, it is useful to compare our findings with prior studies in terms of the exploration and exploitation activation regions. Basically, our findings are similar to those of prior studies. For the exploration orientation, the middle frontal gyrus within dlPFC together with the IPL form the central executive network (CEN)^32,33^. They engage in information retention and manipulation during working memory, problem solution and goal-oriented decision-making^34–36^. In line with previous studies^37,38^ dACC was also activated. CEN coupling with anterior insula and dACC comprise the putative cognitive control network^39–41^. Since dACC links to reasoning centers in the frontal lobe and the memory centers in the limbic system, its activation is understandable when people are switching from habitual exploitation to a novel exploration selection. Furthermore, anterior insula coupling with dACC form the major components of the salience network, implying the subjective salience of external stimuli contributing to complex cognitive processes such as the central executive function as well as affective processes^42^. Collectively, these results are in high conformity with the previous findings of Daw et al.^13^ and Laureiro-Martãnez et al.^4^, suggesting that the four-arm bandit task is valid and effective in probing brain regions associated with exploration, even in an Asian cultural setting.

Neuroscientists have identified several brain regions associated with exploration and have made some progress toward identifying those regions^4,43^. Relevant literature suggests that switching from exploitation to exploration requires individual decision makers to constantly monitor the reward and costs associated with exploitative and explorative activities. According to Koechlin and Hyafil^38^, the FPC, the most anterior part of the frontal lobes, forms the apex of the executive system underlying decision-making^38^. These two scholars found that FPC contributes to human cognitive learning and exploration. Especially, the FPC is robustly engaged when subjects are instructed to learn new behavioral routines. FPC activity specifically correlates with the amount of uncertainty with multiple options. Their study shows that FPC is active whenever subjects depart from an a priori optimal option to check alternative ones. FPC may also play a critical role in the gradual formation of complex behavioral and cognitive routines. Daw et al.^13^ found that FPC and IPL are preferentially active during exploratory decisions; by contrast, regions of vmPFC exhibit activity characteristic of an involvement in value-based exploitative decision-making^13^.

We acknowledge that there are several limitations of this study. First, the homogeneity of the participants is a concern. Although our participants are from different colleges of the same university, in general they have a similar orientation going through a fierce entrance examination competition. Second, our participants are all college students. In the future, recruiting both managers (experts) and students (beginners) for comparison may yield more fruitful results. For future research, recruiting more participants with both high exploration and exploitation scores will enable us to study this particular group for more insights, as they are the type of person businesses are looking for.

To our understanding, this research is the first study using fMRI to evaluate the association between BAS/BIS orientation and explorative decision-making performance. The implications of our research findings include: first, the fMRI result using the four-armed-bandit to test exploration is robust, irrespective of research done in the west or east, and with managers or students. Second, individuals who receive low performance in explorative decision-making have higher BAS scores and make more explorative attempts. They tend to rely less on systematic searching in making decisions when encountering uncertain situations. Third, the BAS/BIS scores of the low-performance group have significant association with their neural activations during explorative decision-making. In particular, their BAS is negatively associated with neural networks related to reward-seeking, and their BIS is negatively associated with neural networks responding to risk-choice. This study provides both behavior- and neuroimaging-based evidences that BAS/BIS scores are associated with mental activation during explorative decision-making for those with poor explorative decision-making skills. The scores may be an effective predictor for the personal motivational orientation of an approach task; moreover, the enhanced mental activity of the high-performance group may lead to their effective exploration and superior exploration performance. Our results have provided a novel insight into understanding the correlation between the behavior motivational scale and mental motivational systems in explorative decision-making.

Our findings have useful practical implications. Specifically, compared with the high-performance group, the low-performance group with a high BAS score does not have the expected neural control in their brain activation, which may indicate blind exploration attempts. One important implication for management development is the emphasis on calculated risk-taking considering reward-seeking at the same time for exploration effectiveness. Those who are bold enough but attempt to try every opportunity without exercising high level cognitive control and analysis, may not be the type of persons that organizations would like to hire. In summary, our study showed that people with relative low BAS scores (associated to reward-seeking network), and relative high BIS scores (associated to risk-choices responding network) may try fewer anticipated low-performance attempts to gain more reward. Thus, they would potentially perform better and gain reward more effectively in explorative decisions making under uncertainty. Hopefully, the results of this study may shed some light on nurturing managers who can better cope with the future viability, dare to explore in an effective way, thus result in superior exploration performance.

## Methods

Before describing our methods in details, we would like to make the following statements.

1. This research has been approved by “Research Ethics Committee of National Chengchi University,” endorsed by Taiwan Ministry of Science and Technology.
2. We confirm that all the methods reported hereunder, including experiments were performed in accordance with relevant guidelines and regulations.
3. We confirm that informed consent was obtained from all participants
4. Our participants’ names and any other information that could lead to the identification of a participant have been removed from all sections of the manuscript, including supplementary information.

### Participants

We recruited 112 students (potential managers) from a university in this fMRI study, and later discarded one participant due to head motion exceeding the threshold (translation in any direction > 1 voxel). The functional data of the remaining 111 participants (37 males, 21.8 ± 1.8 years-old) were included in our analysis. All participants were right-handed, with no metal devices in their bodies (such as dental braces), with no history of psychiatric or neurological disorders, and who were not under psychoactive medications. They all gave written consent to undertake the experimental procedure, which was approved by the Research Ethics Committee of National Chengchi University.

### Behavioral assessment

We assessed participants’ BAS and BIS status using the Taiwan version of BAS/BIS assessments, which was translated and modified by Lee^44^. Factor analysis of the original 20 items (excluding 4 filler items) in Lee’s study showed that BAS-RR item #18 (when a good thing happens to me, it affects me strongly) was unexpectedly loaded on the BIS factor (0.45). Similarly, Müller and Wytykowska also found unexpected loading of the same item on the BIS factor^45^. After removing this item, resulting in a 19-item structure, the internal consistency of BAS-Taiwan was 0.81 and that of BIS-Taiwan was 0.76. The coefficients of test–retest reliability were 0.65 and 0.78, respectively. The 19-item structure was used to assess participants’ BAS and BIS in this study.

### Experimental procedure

Following the design of Daw et al.^13^ and Laureiro-Martinez et al.^6^, participants engaged in a “four-armed bandit” task for which they had to choose among slots. This task captures the key elements of various settings in which individuals are faced with the problem of choosing among options with uncertain outcomes^6,46^. This feature allowed us to study exploratory decisions under uniform conditions in the context of a single task^6,13^.

**Figure 5:**
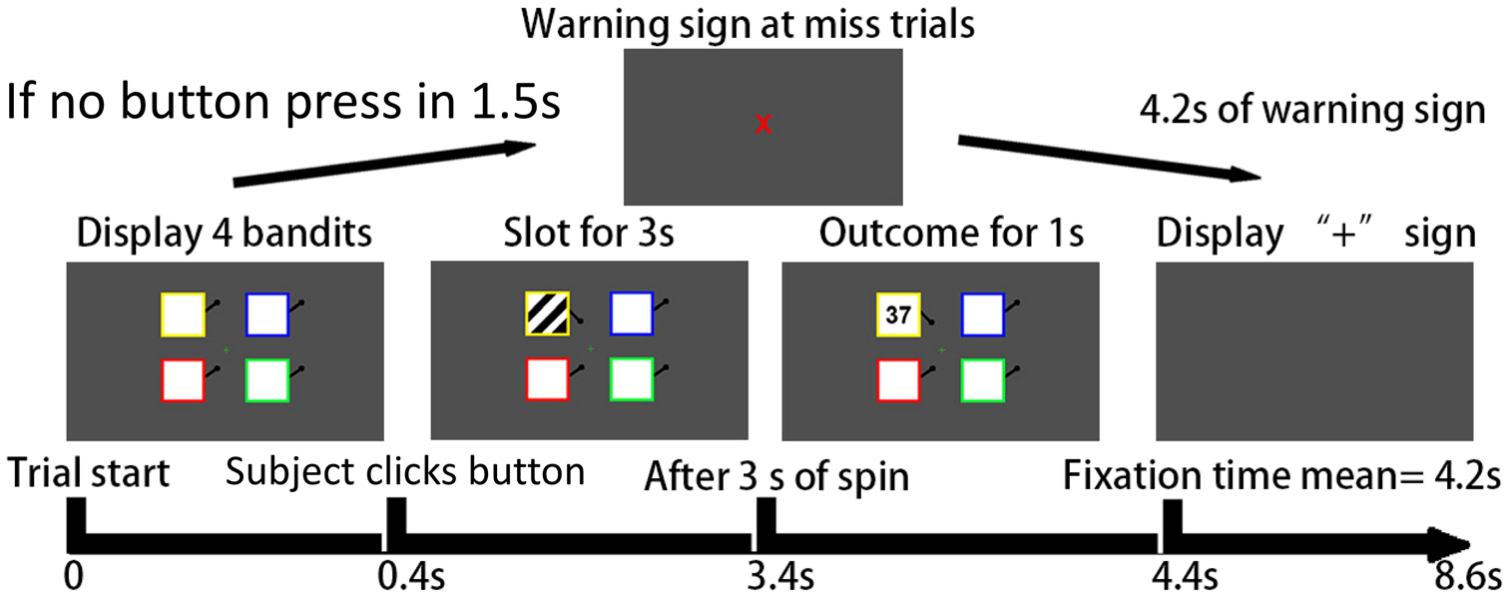
Experimental paradigm. Initially, four slots are presented. The participant chooses one, which then spins. Three seconds later the number of points won is revealed. After a further second the screen is cleared. The condition was set as fail if the participant did not choose one slot within 1.5 s. The next trial was triggered after a fixed trial length of 8.6 s. There were 75 trials of four-armed bandit task in one session, and 300 trials were applied to each participant totally.

The game makes use of four slot machines that pay off points randomly, with the presentation changing from trial to trial (Fig. 5). Participants chose one by pressing one of the four buttons on a keyboard. Within a few seconds, the number of points they had won was displayed, and then a fixation cross appeared, signaling the end of one trial and the beginning of a new one. Subjects had a maximum of 1.5 s to make their choice; if no choice was made within the slots-presentation period, a red X-character was displayed for 4.2 s, and a new trial was started. Participants played a total of 300 trials divided into four sessions (75 trials each). After each session, they were given a break for as long as they wanted.

During each trial, participants had to resolve the dilemma of whether to choose an uncertain but familiar option (exploitation) or investigate a new one in the hope of a higher payoff (exploration). This study adopted the model of Daw et al.^13^ by classifying trials according to whether the actual choice was the one predicted by the model to be the dominant slot machine with the highest expected value (exploitative) or a dominated machine with a lower expected value (exploratory).

### Imaging acquisition

For imaging data collection, participants were scanned using a 3T MR system (MAGNETOM Skyra, Siemens, Erlangen, Germany) and an 8-channel array head coil at the Taiwan Mind and Brain Imaging Center, National Chengchi University. In the functional scanning, thirty-two slices of axial images were acquired using a gradient echo planar imaging (EPI) with the following parameters: TR = 2000 ms, TE = 30 ms, flip angle = 83°, in-plane field of view = 256 × 256 mm, slice thickness = 4.0 mm and acquisition matrix = 64 × 64 × 34 to cover the whole cerebrum. A Magnetization Prepared Rapid Gradient Echo T1-weighted image with spatial resolution of 1 mm isotropic (TR = 2000, TE = 2.3, flip angle = 8°, matrix size = 256, field of view = 240, number of slices = 192) was acquired for each participant.

### fMRI data preprocessing and analysis

We used SPM12 statistical parametric mapping software (Wellcome Trust Centre for Neuroimaging; http://www.fil.ion.ucl.ac.uk/spm) and the Artifact Detection Tools (ART) toolkit for preprocessing and statistical analysis of the fMRI data. The first eight volumes of each run were discarded to exclude T_1_ saturation effects. Then the functional images were corrected for differences in slice-acquisition time to the first volume and were realigned to the first volume by the ART toolkit. By using ART, an image was considered as an outlier if the head displacement was greater than 4 mm in any plane, or if the rotational displacement was greater than 1°. Individual anatomical images were registered into the realigned images, and all co-registered images were normalized to the standard MNI (Montreal Neurological Institute) average template and resampled to a 2-mm isotropic voxel size. Normalized images were spatially smoothed with a Gaussian kernel of 6-mm full width at half maximum (FWHM) to accommodate any anatomical variability across participants.

Two conditions were utilized in the experiment: exploitation and exploration. The onset time of each event was set at the start of a slot presentation. Data from each participant were entered into a general linear model using an event-related designed procedure. The two conditions in this study were modeled using a box-car function convolved with a canonical hemodynamic response function. Six motion parameters were adopted as the nuisance covariate factors and were considered in the individual analysis. Group-level results of exploitative and explorative decision-making were acquired using one-sample t-test. Participants’ BAS/BIS scores were used as covariates in a one-sample t-test to investigate a high level of behavior-orientation neural activity. To test why some individuals are good at making explorative decisions, categorical data analysis was applied into all the behavior and functional data. All participants were then separated into 2 groups based on their median score during explorative decision-making to investigate the impact of the two groups. The resulting model coefficients for individual participants were subjected to a subsequent 2-sample t-test with participants’ BAS/BIS scores as covariates. All activity was thresholded at Alpha-shim corrected *p* < 0.05 by AFNI software^47^ for multiple comparison correction.

## Supplementary Information

Supplementary Legends.

Supplementary Figure S1.

Supplementary Figure S2.

Supplementary Figure S3.

Supplementary Figure S4.

Supplementary Figure S5.

## References

[1] March JG (1991). Exploration and exploitation in organizational learning. Organ. Sci..

[2] O'Reilly CA, Tushman ML (2004). The ambidextrous organization. Harvard Business Rev..

[3] Lavie D, Stettner U, Tushman ML (2010). Exploration and exploitation within and across organizations. Acad. Manag. Ann..

[4] Laureiro-Martínez D, Canessa N, Brusoni S, Zollo M, Hare T, Alemanno F, Cappa SF. Frontopolar cortex and decision-making efficiency: comparing brain activity of experts with different professional background during an exploration-exploitation task. *Front Hum Neurosci.* **7**, 927 (2014). 10.3389/fnhum.2013.00927. PMID: 24478664; PMCID: PMC3897871. 10.3389/fnhum.2013.00927 PMC3897871 24478664

[5] Levinthal DA, March JG (1993). The myopia of learning. Strateg. Manag. J..

[6] Laureiro-Martínez D, Brusoni S, Canessa N, Zollo M (2015). Understanding the exploration–exploitation dilemma: An fMRI study of attention control and decision-making performance. Strat. Manag. J..

[7] Piao M (2014). A long life after exploitation and exploration. Eur. J. Innov. Manag..

[8] Raisch S, Birkinshaw J, Probst G, Tushman ML (2009). Organizational ambidexterity: Balancing exploitation and exploration for sustained performance. Organ. Sci..

[9] Schutte I, Kenemans JL, Schutter DJLG (2017). Resting-state theta/beta EEG ratio is associated with reward- and punishment-related reversal learning. Cognit. Affect Behav. Neurosci..

[10] Kang E, Uhlenbruck K (2006). A process framework of entrepreneurship: From exploration, to exploitation, to exit. Acad. Entrepreneurship J..

[11] Carver CS, White TL (1994). Behavioral inhibition, behavioral activation, and affective responses to impending reward and punishment: The BIS/BAS Scales. J. Person. Social Psychol..

[12] Baker DF, Larson LM, Seipel MT (2017). Relation of reinforcement sensitivity on vocational interest and self-efficacy. J. Career Assess..

[13] Daw ND, O'Doherty JP, Dayan P, Seymour B, Dolan RJ (2006). Cortical substrates for exploratory decisions in humans. Nature.

[14] Krug A (2014). Investigation of decision-making under uncertainty in healthy subjects: A multi-centric fMRI study. Behav. Brain Res..

[15] Li C-W, Chen J-H, Tsai C-G (2015). Listening to music in a risk-reward context: The roles of the temporoparietal junction and the orbitofrontal/insular cortices in reward-anticipation, reward-gain, and reward-loss. Brain Res..

[16] Sidlauskaite J (2014). Anticipatory processes in brain state switching—Evidence from a novel cued-switching task implicating default mode and salience networks. Neuroimage.

[17] Uddin LQ (2015). Salience processing and insular cortical function and dysfunction. Nat. Rev. Neurosci. 2009 10:6.

[18] Fan L (2014). Connectivity-based parcellation of the human temporal pole using diffusion tensor imaging. Cereb. Cortex.

[19] Bai T (2018). Decreased connection between reward systems and paralimbic cortex in depressive patients. Front. Neurosci..

[20] Tobler PN, O'Doherty JP, Dolan RJ, Schultz W (2007). Reward value coding distinct from risk attitude-related uncertainty coding in human reward systems. J. Neurophysiol..

[21] Cho C, Smith DV, Delgado MR (2016). Reward sensitivity enhances ventrolateral prefrontal cortex activation during free choice. Front. Neurosci..

[22] Hsu M, Bhatt M, Adolphs R, Tranel D, Camerer CF (2005). Neural systems responding to degrees of uncertainty in human decision-making. Science.

[23] Cole MW, Schneider W (2007). The cognitive control network: Integrated cortical regions with dissociable functions. Neuroimage.

[24] Naqvi NH, Bechara A (2009). The hidden island of addiction: The insula. Trends Neurosci..

[25] Menon V, Uddin LQ (2010). Saliency, switching, attention and control: A network model of insula function. Brain Struct. Funct..

[26] Hopf J-M (2004). Popout modulates focal attention in the primary visual cortex. Neuroimage.

[27] Smith AT, Cotillon-Williams NM, Williams AL (2006). Attentional modulation in the human visual cortex: The time-course of the BOLD response and its implications. Neuroimage.

[28] Silver MA, Ress D, Heeger DJ (2007). Neural correlates of sustained spatial attention in human early visual cortex. J. Neurophysiol..

[29] Ciaramitaro VM, Buracas GT, Boynton GM (2007). Spatial and cross-modal attention alter responses to unattended sensory information in early visual and auditory human cortex. J. Neurophysiol..

[30] Spooner RK, Wiesman AI, Proskovec AL, Heinrichs-Graham E, Wilson TW (2019). Prefrontal theta modulates sensorimotor gamma networks during the reorienting of attention. Hum. Brain Mapp..

[31] Zorowitz S (2019). The neural basis of approach-avoidance conflict: A model based analysis. eNeuro.

[32] Seeley WW (2007). Dissociable intrinsic connectivity networks for salience processing and executive control. J. Neurosci..

[33] Menon V. Salience Network. In: Arthur W. Toga, editor. Brain Mapping: An Encyclopedic Reference, vol. 2, pp. 597–611. Academic Press: Elsevie (2015).

[34] Miller EK, Cohen JD (2003). An integrative theory of prefrontal cortex function. Annu. Rev. Neurosci..

[35] Petrides M (2005). Lateral prefrontal cortex: Architectonic and functional organization. Philos. Trans. R. Soc. B Biol. Sci..

[36] Rottschy C (2012). Modelling neural correlates of working memory: A coordinate-based meta-analysis. Neuroimage.

[37] Aston-Jones G, Cohen JD (2005). An integrative theory of locus coeruleus-norepinephrine function: Adaptive gain and optimal performance. Annu. Rev. Neurosci..

[38] Koechlin E, Hyafil A (2007). Anterior prefrontal function and the limits of human decision-making. Science.

[39] Wager TD (2005). Common and unique components of response inhibition revealed by fMRI. Neuroimage.

[40] Levy BJ, Wagner AD (2011). Cognitive control and right ventrolateral prefrontal cortex: Reflexive reorienting, motor inhibition, and action updating. Ann. N.Y. Acad. Sci..

[41] Cai W (2016). Causal interactions within a frontal-cingulate-parietal network during cognitive control: Convergent evidence from a multisite–multitask investigation. Cereb. Cortex.

[42] Chang T-T, Lee P-H, Metcalfe AWS (2018). Intrinsic insula network engagement underlying children's reading and arithmetic skills. Neuroimage.

[43] Boorman ED, Rushworth MFS (2009). Conceptual representation and the making of new decisions. Neuron.

[44] Lee, Y. P. Exploring attention of individuals with depressed mood: The moderating roles of behavioral inhibition and activation system. Unpublished Master’s Thesis. National Taiwan University, Taipei, Taiwan 1–80 (2010). 10.6342/NTU.2010.00413.

[45] Müller JM, Wytykowska AM (2005). Psychometric properties and validation of a Polish adaptation of Carver and White’s BIS/BAS scales. Personal. Individ. Differ..

[46] Meyer RJ, Shi Y (1995). Sequential choice under ambiguity: Intuitive solutions to the Armed–Bandit problem. Manage. Sci..

[47] Cox RW (1996). AFNI: Software for analysis and visualization of functional magnetic resonance neuroimages. Comput. Biomed. Res..

