# Supplementary material for: Motivational system modulates brain responses during exploratory decision-making
Source: Sci Rep. 2021 Aug 4;11:15810. doi: 10.1038/s41598-021-95311-0 (PMC8339076; doi:10.1038/s41598-021-95311-0)
Supplement: Supplementary file 1 — Supplementary Legends. [file 41598_2021_95311_MOESM1_ESM.docx]

**Supplementary Figures Legends**

**Figure S1. The mental activity during explorative decision making.**

The main activation regions of male participants are similar to those of female participants during **explorative** decision making.

**Figure S2. The mental activity during exploitative decision making.**

The main activation regions of male participants are similar to those of female participants during **exploitative** decision making.

**Figure S3. The different activation while comparing the explorative decision making to exploitative decision making.**

The main regions showed significant difference between explorative and exploitative decisions-making of male participants are similar to those of female participants.

**Figure S4. The distribution of performance during explorative decision making.**

The groups with extreme higher-performance scores (>50.3) and with lower-performance scores (<47.7) were used for the activation analysis.

**Figure S5. The different mentation activation between extreme high- and low- performance groups during explorative decision making.**

The activation maps showed that the neural activity is similar to the result we present in the main texts (Figure 3).
