# Supplementary figures and images for: Motivational system modulates brain responses during exploratory decision-making
Source: Sci Rep. 2021 Aug 4;11:15810. doi: 10.1038/s41598-021-95311-0 (PMC8339076; doi:10.1038/s41598-021-95311-0)

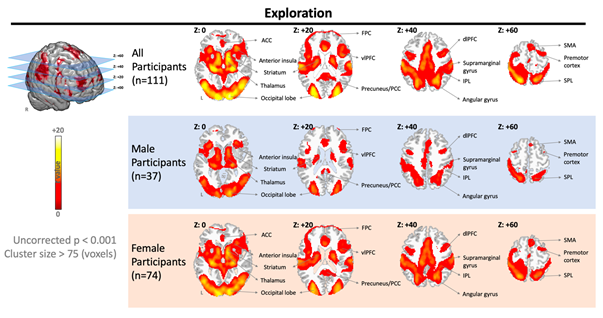

Supplement: Supplementary file 2 — Supplementary Figure S1. [file 41598_2021_95311_MOESM2_ESM.tif]

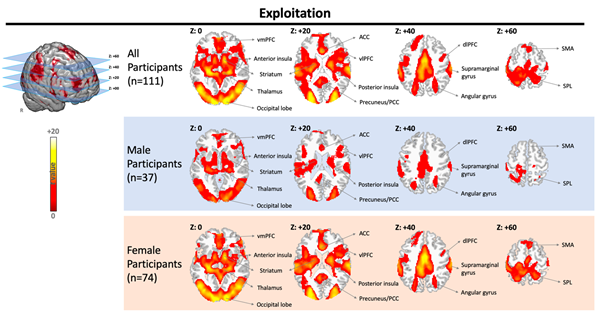

Supplement: Supplementary file 3 — Supplementary Figure S2. [file 41598_2021_95311_MOESM3_ESM.tif]

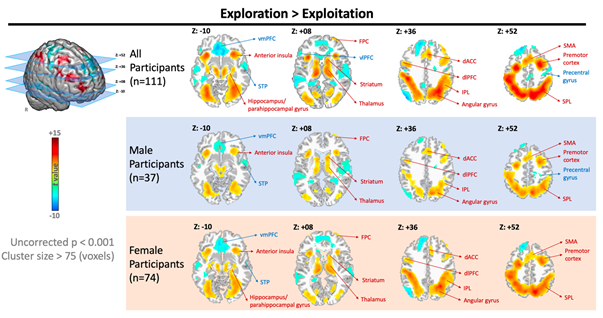

Supplement: Supplementary file 4 — Supplementary Figure S3. [file 41598_2021_95311_MOESM4_ESM.tif]

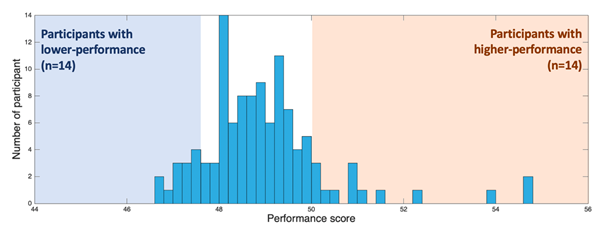

Supplement: Supplementary file 5 — Supplementary Figure S4. [file 41598_2021_95311_MOESM5_ESM.tif]

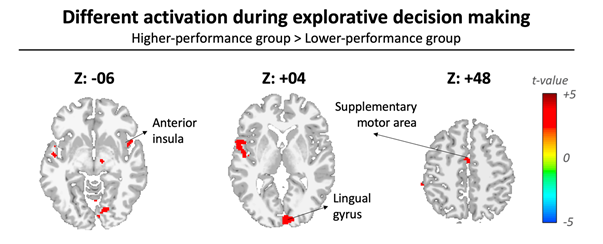

Supplement: Supplementary file 6 — Supplementary Figure S5. [file 41598_2021_95311_MOESM6_ESM.tif]
